# Femoral or axillary artery as a cannulation site in the acute aortic dissection type A surgery: is there still a doubt?

**DOI:** 10.1186/1749-8090-8-S1-O27

**Published:** 2013-09-11

**Authors:** M Matkovic, S Putnik, M Cubrilo, M Grujic, M Samanovic, A Djordjevic, M Zlatkovic, M Ristic

**Affiliations:** 1 Cardiac Surgery, Clinical Center of Serbia, Belgrade, Serbia

## Background

Anterograde perfusion is widely addopted strategy in aortic dissection type A treatement. Physiological blood flow direction reduces malperfusion complications during CPB and allows anterograde cerebral perfusion during circulatory arrest. In spite of these facts, femoral artery is still used as a cannulation site by surgeons worldwide. Primary end point was to analize the mortality in the two groups of patients (anterograde vs retrograde perfusion during CPB). Secondary end point was to compare frequency of postoperative complications, length of stay in ICU and in hospital.

## Methods

We performed a retrospective sudy that included 99 patients which were surgically treated for acute aortic dissection type A in Clinical Center of Serbia from 01.01.2010 to 31.01.2013. Patients were divided into two groups according to the cannulation site for CPB (anterograde vs retrograde CPB). We analized comorbidities, operative parameters, postoperative complications and intrahospital mortality between the two groups of patients.

## Results

In the anterograde perfusion group longer pain to admission and admission to OR period has been observed (p<0,05). Patients in the anterograde group had statistically significant less neurological, renal and respiratory postoperative complications (p<0,05). However there was no statisticaly significant difference between the two groups regarding intrahospital mortality (p<0,05).

## Conclusion

In spite of lower complication rate in the anterograde perfusion group, there is stll not enough evidence of its superiority in every dissection case. The cannulation site in an aortic dissection type A surgery remains a surgeon's preference and should be carefully chosen on a case-by-case basis.

